# Soft Sulphur for Stopping Teeth

**Published:** 1858-07

**Authors:** 


*Soft Sulphur for Stopping Teeth.*—M. Henriot has been in the habit of substituting soft sulphur for the ordinary cements for filling teeth, and he says, that it soon becomes hard and insoluble. He puts in a small glass tube, closed at one end, some fragments of common sulphur, or better still, a little washed sulphur, he heats it over a spirit lamp, and pours it into water. The whole preparation occupies about two minutes. The art consists in hitting the exact temperature, which must be high. The operator may know that he has succeeded, if the sulphur, after its first fusion, has become viscid and then liquid again. If in this state it be thrown into water, it becomes a brown, soft, elastic, spongy mass.

